# A Community-Informed Toolkit for Physician-Scientist Competencies and Milestones: Bridging the Worlds of Medicine and Research

**DOI:** 10.1093/acamed/wvag053

**Published:** 2026-07-01

**Authors:** Lourdes Estrada, Justin T. Baca, Margaret H. Baron, Lawrence F. Brass, Clarissa S. Craft, Jose E. Cavazos, Aimee W. Kao, Mary-Claire Roghmann, Jodi Yellin, Wayne T. McCormack

**Affiliations:** 1 Department of Biochemistry, Vanderbilt University School of Medicine, Nashville, TN, United States; 2 Department of Emergency Medicine, University of New Mexico, Albuquerque, NM, United States; 3 Departments of Medicine, Stem Cell Biology and Regenerative Medicine, and Oncological Sciences, Icahn School of Medicine at Mount Sinai, New York, NY, United States; 4 Departments of Medicine and Pharmacology, University of Pennsylvania Perelman School of Medicine, Philadelphia, PA, United States; 5 Department of Cell Biology and Physiology, Washington University in St. Louis School of Medicine, St Louis, MO, United States; 6 Department of Neurology, University of Texas Health San Antonio, San Antonio, TX, United States; 7 Department of Neurology, Weill Institute for Neuroscience, University of California San Francisco School of Medicine, San Francisco, CA, United States; 8 Departments of Epidemiology and Public Health and Medicine, University of Maryland School of Medicine, Baltimore, MD, United States; 9 Department of Research Workforce, Training, and Science Policy, Association of American Medical Colleges, Washington, DC, United States; 10 Department of Pathology, Immunology, and Laboratory Medicine, University of Florida College of Medicine, Gainesville, FL, United States

**Keywords:** competency-based education, medical education, physician-scientists, curriculum development, professional development

## Abstract

In 2022, the MD-PhD Competencies Development Workgroup, a subgroup of the Association of American Medical Colleges Group on Research, Education, and Training, initiated a project to define essential competencies for training physician-scientists, with a focus on MD-PhD education programs. The primary objective was to develop a comprehensive toolkit containing well-defined competencies and milestone-based tools to guide the education of individuals in MD-PhD combined degree programs. It is intended to augment, not duplicate, existing MD and/or PhD competency rubrics. A systematic approach was adopted in creating the toolkit, which included opportunities for the physician-scientist training community to contribute their collective experience and knowledge. This article describes the toolkit and its 3 main components: (1) 14 competencies grouped in 4 core domains, (2) a milestone-based assessment tool to track learner progress, and (3) guidelines for implementing the toolkit. This framework can be used to align expectations for learners and mentors, assess learner development, facilitate mentor-mentee conversations, adjust individualized learning goals, and self-identify curricular strengths and gaps. The toolkit can serve as a valuable resource for program directors, faculty mentors, and learners, empowering them to collaboratively shape the next generation of physician-scientists, bridging the worlds of medicine and research.

The MD-PhD Competencies Development Workgroup, a subgroup of the Association of American Medical Colleges (AAMC) Group on Research, Education, and Training (GREAT), convened in 2022. This workgroup, composed of members from medical schools across the country, was charged with identifying and documenting the essential competencies—knowledge, skills, and attitudes—specific to physician-scientists. The aim was to establish a comprehensive set of competencies and assessment tools, including developmental milestones, to enhance the training and success of physician-scientists while considering the distinctive objectives of MD-PhD programs.

## Background

Unlike traditional time-based curricula, competency-based education (CBE) focuses on demonstrating specific competencies, allowing learners to progress at their own pace and concentrate on areas that require the most development.^1^ Competency-based medical education (CBME) has become valuable for training future physicians to ensure that learners acquire the necessary knowledge, skills, and attitudes to provide high-quality patient care.^2^ Studies have shown that CBME can improve learner engagement, better align training and practice, enhance patient outcomes, increase learner satisfaction, and better prepare graduates.^3–5^ As the medical education landscape evolves, adopting CBME principles is crucial to producing physicians well equipped to meet the changing needs of the health care system.

CBE has also gained traction in graduate programs, demonstrating that it can improve student engagement, align coursework with practical applications, increase preparedness for professional roles, and provide a stronger sense of agency in their learning.^6,7^ A longitudinal analysis by Zlatkin-Troitschanskaia et al^8^ demonstrated that CBE-trained graduate students exhibited stronger critical thinking and problem-solving abilities than their traditionally educated counterparts.^8^ Verderame et al^9^ proposed a milestone-based framework for science PhD training, which defined 44 competencies in 10 domains, with accompanying milestones for predoctoral through postdoctoral training stages. Educators in the Clinical and Translational Science Award workforce development community have taken an interest in CBE for leadership, mentoring, team science, regulatory science, and clinical research.^10–16^

## The Gap in Physician-Scientist Education

Despite CBE’s success in medical and graduate education, physician-scientist training lacks a comparable framework. Physician-scientists bridge clinical practice and biomedical research, yet traditional programs often overlook the competencies needed for this dual role.^17^ A competency-based approach could fill this gap by fostering skills essential to physician-scientists—identifying knowledge gaps, translating discoveries into patient care, and developing research questions that address unmet health needs.^18,19^ As the demand for physician-scientists continues to increase, incorporating CBE principles into training could foster the development of a cadre of physician-scientists uniquely positioned to drive progress at the intersection of medicine and science.

## Development of the MD-PhD Competencies Toolkit

The workgroup initiated its process by examining the existing competencies for MD students^2–5^ and the recently established competencies for PhD students.^9^ The workgroup identified gaps and proposed a set of competencies across 4 distinct domains (Figure 1), which were presented at the AAMC GREAT National Conference in 2022 and 2023 to solicit input, feedback, and recommendations from MD-PhD program directors and administrators. In 2024, they held listening sessions with MD-PhD students, MD-PhD program directors, and department chairs to discuss the proposed competencies, identify any remaining gaps, understand their added value, and secure their support.

With the domains and competencies established, the workgroup crafted milestones (ie, brief descriptions of observable behaviors) for each competency at 5 levels of skill acquisition: (1) novice (entry-level student), (2) advanced beginner (MD-PhD training), (3) competent (graduation and entry to residency), (4) proficient (residency and fellowship), and (5) expert (physician-scientist).^9,20^ The proposed milestones were refined through iteration until a consensus was reached and presented at the AAMC GREAT National Conference in 2024.

The workgroup chose a community-engaged approach to encourage acceptance and implementation of the competencies and increase the likelihood that the toolkit would be adopted. This was accomplished through a mixed-methods data collection approach, integrating quantitative data from surveys conducted before and after the workshops and qualitative insights from listening sessions. The presession surveys assessed baseline knowledge, skills, and attitudes toward physician-scientist roles, whereas the postsession surveys measured changes and outcomes attributable to the workshop session. Qualitative data (observations and recorded responses) provided essential input from the toolkit’s target audiences in the design process.

## Core Competency Domains

The workgroup established guiding principles for creating MD-PhD competencies to ensure they were effective, efficient, and feasible. These principles were to (1) focus on competencies that bridge both medicine and research, (2) limit the total number of competencies to reduce cognitive load and implementation logistics, (3) minimize overlap with competencies that are exclusive to either MD or PhD programs, and (4) incorporate competencies that align with the principles of health and research equity. With these principles in mind, we sought feedback from the MD-PhD community on the preliminary domains. As a result, 4 distinct core competency domains emerged (Figure 1).

The first core competency domain was research to health, which is centered on applying clinical insights to research endeavors and vice versa, ensuring a seamless integration of medical practice with scientific investigation. The second core competency domain was communication skills, which highlights the need for clear and effective communication tailored to audiences such as fellow clinicians and researchers, funding agencies, and the public. The third core competency domain was professional growth, which emphasizes fostering essential skills and knowledge for physician-scientist career progression, such as mentorship, a commitment to ongoing learning and curiosity, and the ability to collaborate effectively with others in cross-disciplinary teams. The fourth core competency domain was personal growth, which focuses on personal development and maintaining mental health to encourage resilience, coping with stress and avoiding burnout, understanding one's identity as a physician-scientist, and recognizing and addressing personal biases.

The specific competencies outlined in Table 1 under the main competency domains capture the essential skills that physician-scientists should acquire during their dual-degree training. For instance, in the research to health domain, competency A emphasizes the importance of using scientific and clinical knowledge to identify gaps and unmet health needs and to address problems in clinical and research environments. To ensure that research benefits everyone, we incorporated health equity principles into clinical practices and research programs to reduce inequities.

The communication domain is particularly relevant to physician-scientists, who are uniquely positioned to bridge communication with many audiences. Therefore, the workgroup and stakeholders emphasized the importance of conveying research findings to clinicians, discussing their implications for clinical practice, communicating clinical findings to scientists, and exploring their relevance to research.

In the domains of professional and personal growth, we focused on skills that foster persistence in the physician-scientist career path. These skills include mentoring, navigating clinical and research environments (eg, time management to balance clinical and research responsibilities), collaborating in interprofessional teams, developing a professional identity, and managing burnout.

The workgroup intentionally sought to minimize overlap with competencies unique to either MD or PhD programs. However, the final toolkit includes several competencies relevant to both groups (eg, research to health [competencies B and C], professional growth [competency B], and personal growth [competencies A and D]). This overlap reflects the integrated nature of the physician-scientist career, which requires skills that cannot be neatly separated. After extensive discussion with community stakeholders, the workgroup concluded that the developmental milestones capture the distinctive perspective of physician-scientists. For example, competency B in professional growth applies broadly to all professionals, but the milestones illustrate a trajectory specific to physician-scientists, emphasizing the integration of research and clinical expertise, openness to new ideas across both settings, and the ability to apply evidence and experience to inform career choices.

In addition to trainee self-assessment, this toolkit should be used by individuals who directly observe trainees demonstrating the specified behaviors. Milestones are most effectively applied by assessors with firsthand knowledge of a trainee’s performance. In MD-PhD programs, these individuals may include clinical preceptors, research mentors, research collaborators, thesis committee members, peers, near-peers (residents, fellows, or physician-scientist training program trainees), and MD-PhD program directors.

## Benefits of the Toolkit

The MD-PhD Competencies Toolkit stands out for its modular design, which facilitates seamless integration into activities tailored to the programs’ distinct needs, objectives, and priorities. The toolkit may help foster growth through self-assessments, structured mentor-mentee dialogues, and formative feedback in the learning environment. By assisting in the creation of personalized learning objectives and development plans, the toolkit plays a critical role in shaping learners' educational journeys. Additionally, it may be valuable for guiding curricular design by ensuring that academic content is relevant and aligned with the desired learning outcomes.

Competency-based assessments transform student learning by clearly defining the essential skills and knowledge learners must acquire. Unlike traditional assessments that emphasize time-based metrics, this approach prioritizes skill mastery, allowing learners to progress as they demonstrate proficiency. This shift promotes individualized learning, focusing on mastering competencies rather than adhering to a one-size-fits-all timeline.

Competency-based assessments provide learners with clear expectations, which are key to promoting efficient learning. A transparent set of goals and criteria demystify the learning process and empower students to take charge of their education. Educators can use assessment data to inform the design of educational strategies and interventions that foster an environment of continuous improvement.

A critical use of the toolkit is to support mentoring conversations focused on key competencies for the MD-PhD scientist. For example, the assessment can be completed independently by mentees and mentors, and the results can be combined into a single report that reveals differences in ratings (Figure 2). The toolkit can identify competencies for which a mentee and a mentor agree (A) or disagree (B and C) about the milestones the mentee has mastered. For some competencies, the mentor may indicate that the mentee has mastered an additional milestone, suggesting that the mentee may be underconfident in that skill (B). Alternatively, a mentee may indicate mastery for a higher milestone than the mentor, suggesting that the mentee may be overconfident or that the mentor has not observed that behavior. Most importantly, the goal is to stimulate discussion focused on the competencies and on why each milestone was selected, leading to a better understanding of the mentee’s competency level and enabling the prioritization of next training steps to achieve growth. Learners should be encouraged to regularly refer to the toolkit to reflect on their progress during MD-PhD, residency, and fellowship training, establish personal objectives, and identify ways to fill gaps in their competencies. Mentors and sponsors with experience as physician-scientists could use the toolkit as a basis for discussion in their regular meetings with learners.

The toolkit can facilitate personalized learning by tailoring educational pathways to learners’ unique skills and interests. By leveraging this method, learners can collaborate with mentors to identify areas for growth and set personalized goals that are both challenging and achievable. Tools such as the National Institutes of Health Individual Development Plan (IDP) can serve as a framework to help guide this process because it primarily focuses on learner-unguided self-reflection on their progress and career objectives. The integration of the IDP with the MD-PhD Competencies Toolkit would foster a more active and guided approach to professional development and self-reflection, ensuring that all domains are addressed. This targeted approach enhances engagement and motivation, ensuring that progress aligns continuously with both immediate learning needs and long-term career aspirations, thereby making the training experience more efficient and effective.

Pooled competency-based assessment data can facilitate program evaluation by identifying milestones that learners are meeting or not at appropriate stages of physician-scientist training. Program evaluation supports enhancing physician-scientist training by systematically assessing the effectiveness of educational activities and identifying areas for improvement. By using rigorous evaluation methods, educators and administrators can ensure that the curriculum aligns with the evolving demands of health care and research and is responsive to the needs of emerging physician-scientists.^21^ Through continuous evaluation, training programs can adapt to the rapid advancements in medical knowledge and technology, equip learners with the necessary skills to lead innovative research, and provide exemplary patient care.^22^ The toolkit can facilitate program evaluation and improvement by aligning goals with program objectives and driving data collection to ensure learning, behaviors, and outcomes. The evidence-based insights gained through regular feedback can inform policy and process decisions, guiding the allocation of resources toward the most impactful educational strategies.

Typically, the curricula of MD-PhD programs, including but not limited to medical scientist training programs, integrate the curricula of the MD program and PhD programs. Each MD-PhD–specific training element is intentionally designed to impart necessary competencies to learners, focused on the program’s training objectives. The MD-PhD Competencies Toolkit provides a roadmap for these unique skills, which center on the physician-scientist dual lens.^19^ For example, a clinical case studies course to preserve clinical reasoning skills during graduate studies can be modified to consider how research can address gaps in clinical diagnosis, management, and/or treatment (domain 1, competency A in Table 1). Such a course could involve a case discussion led by a final-year MD-PhD student in which participants brainstorm what is currently unknown about a particular disease and how research can help fill those knowledge gaps. Another example is a physician-scientist seminar that showcases how physician-scientists integrate their medical and research training into their daily work as they advance in their careers (domain 3, competency A in Table 1).

## Challenges and Solutions

Implementing CBE and competency-based assessments in MD-PhD programs poses distinct challenges given the intricate and extensive training already involved. One issue raised is that the deluge of detailed milestones required for MD and PhD training can cause cognitive overload.^23^ To address this challenge, our workgroup recommends a toolkit tailored to the unique skills of MD-PhD students, streamlining the number of domains, competencies, and milestones to those most critical to a physician-scientist's career.

Another concern is that milestones might be too specific and restrictive, such as the limited scope of a 5-level scale. The workgroup believes that the MD-PhD Competencies Toolkit complements other assessment methods (eg, simulations, standardized examinations, thesis and promotions committees) to provide a holistic evaluation without unnecessary overlap.

Introducing competency-based assessments into already demanding MD-PhD programs raises concerns about increasing academic burnout and detracting from the learning experience. To mitigate this risk, the toolkit’s modular design allows customization to align with individual educational and career goals while minimizing additional strain. Programs can combine competencies from MD-PhD, PhD, and translational science toolkits to create tailored frameworks that fit their specific needs. The toolkit’s adaptability for online or app-based platforms can facilitate tracking and reduce the burden on learners, assessors, and program administrators, much like an activity tracker provides real-time updates and progress reports. Use of Research Electronic Data Capture (REDCap) or similar database platforms can greatly streamline the implementation of milestone-based assessments. Some programs, for example, have used REDCap to collect structured peer and faculty feedback on trainee presentations, reducing administrative workload and enabling more efficient program evaluation. This approach also facilitates analysis of learning outcomes, professional development, and overall program effectiveness. The IDP can also incorporate milestone frameworks to guide self-assessment and mentor evaluation. Embedding milestones into the IDP promotes structured conversations about progress, goals, and professional growth, creating an integrated system for monitoring and supporting trainees. The fields of graduate and medical education are exploring the use of artificial intelligence (AI) to promote learning.^24,25^ Integrating AI with milestone assessments may enable more personalized, efficient, and adaptive educational experiences for physician-scientist trainees.

An interesting question is whether a competency-based framework for physician-scientist education in MD-PhD programs could shorten or lengthen the time required to complete the degrees. Although medical education has successfully used this approach to streamline MD curricula,^26,27^ there is currently no evidence to suggest that the same benefits apply to MD-PhD programs.

We recognize that not all physician-scientists are trained in MD-PhD programs and suggest that the same competencies apply to other physician-scientists (eg, MD-only) at whatever stage they learn research skills. The milestones are not necessarily linked to career stages. Rather, they are intended to represent a continuum of physician-scientist competency development that might begin as a fellow or early-stage investigator.^24,25^ Despite the challenges of introducing CBE and assessments into MD-PhD programs, our workgroup is optimistic that the strategic use of technology and personalized tools can promote adoption without imposing undue pressure on students or faculty.

## Conclusions

To attempt to transform physician-scientist education, the MD-PhD Competencies Development Workgroup developed essential competencies to enhance the training and success of these crucial contributors to medical research and clinical care. We aimed to address a longstanding gap by creating a comprehensive, competency-based framework tailored to the needs of physician-scientists. The transformative power of CBE has been demonstrated in medical and graduate education, shifting from traditional time-based curricula to a model emphasizing mastery of specific competencies. This change has yielded numerous benefits, including improved outcomes, increased learner engagement, and better alignment between educational efforts and practical applications.

Against this backdrop of proven success, the workgroup’s initiative to extend CBE to physician-scientist education outlines competencies and milestones across 4 key domains. The new framework was developed from the collective wisdom and experience of the MD-PhD community to ground it in practicality. The resulting MD-PhD Competencies Toolkit is a testament to the power of community engagement. It is a versatile resource for training programs, adaptable to fit the unique needs of various educational environments.

As future directions, we envision the toolkit expanding beyond a collection of competencies to catalyze mentor-mentee relationships and personalized learning and assessment. We hope it empowers learners to navigate their educational journey with clarity, enabling them to progress through a learning experience that provides a clear and transparent path to success. As training programs adopt this toolkit, they may also find it valuable for program evaluation to ensure that activities remain aligned with the ever-evolving demands of health care and research, producing well-prepared physician-scientist leaders in innovation and patient care.

We recognize that implementing CBE and assessments presents distinct challenges and are aware of the risks associated with cognitive overload and academic burnout. In response, we encourage training programs to tailor the modular toolkit to simplify and streamline the learning process. We envision the strategic use of technology as essential to unlocking the full potential of CBE and assessment, reducing administrative burdens, and enabling a more personalized educational experience. In summary, we hope that the MD-PhD Competencies Toolkit represents a pivotal advancement in physician-scientist education, reflecting the dynamic and multifaceted nature of their immense value in improving human health.

## Figures and Tables

**Figure 1:**
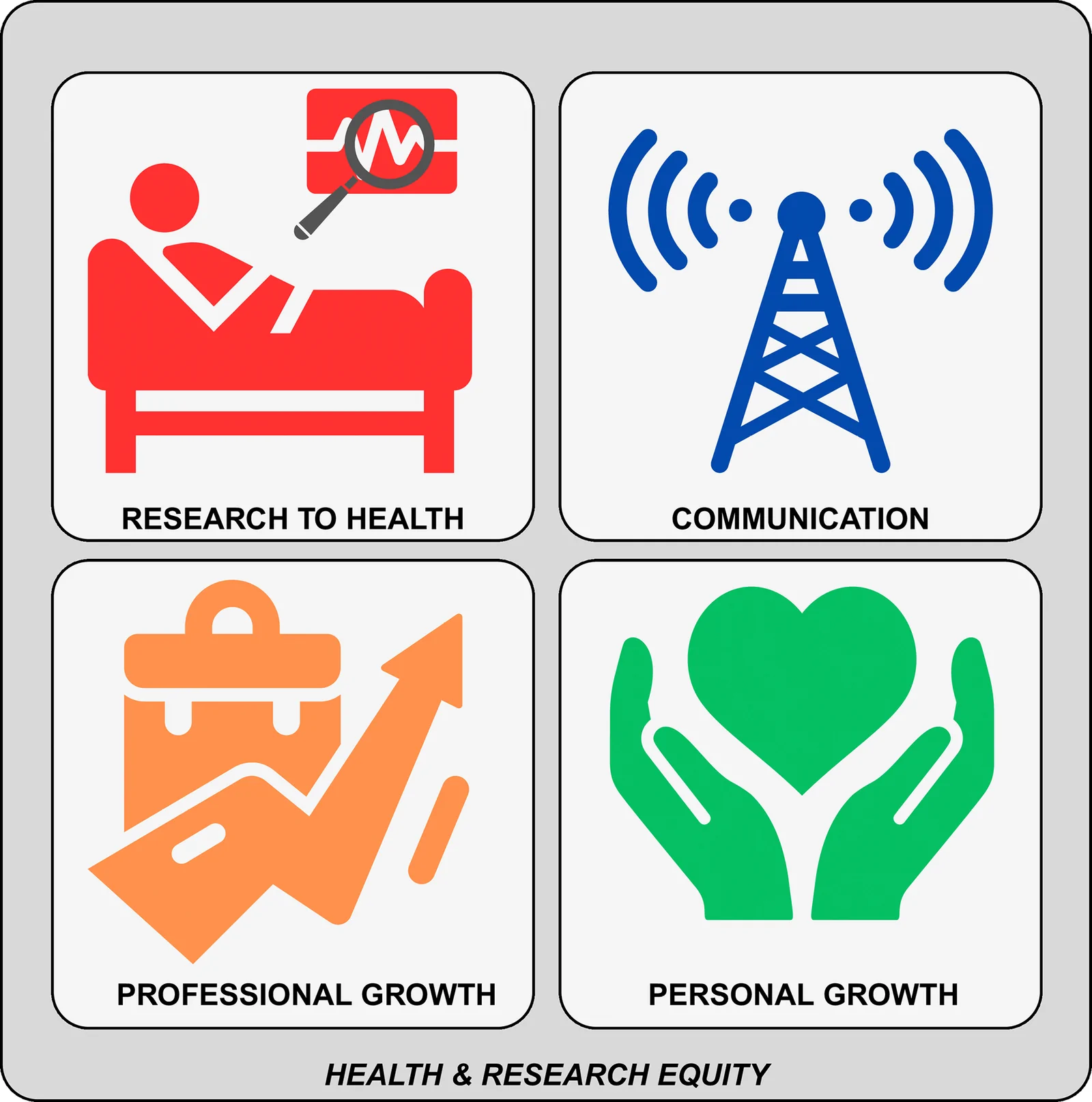
Core domains of the MD-PhD Competency Toolkit. In 2022, the MD-PhD Competencies Development Workgroup, part of the Association of American Medical Colleges Group on Research, Education, and Training, identified 4 interrelated domains essential for physician-scientist training: research to health (emphasizing the integration of clinical insight and scientific discovery), communication (underscoring effective information exchange across diverse audiences), professional growth (highlighting career development, mentorship, and lifelong learning), and personal growth (promoting resilience, well-being, and self-awareness). All domains are grounded in health and research equity, ensuring that scientific and clinical advances benefit all populations.

**Figure 2:**
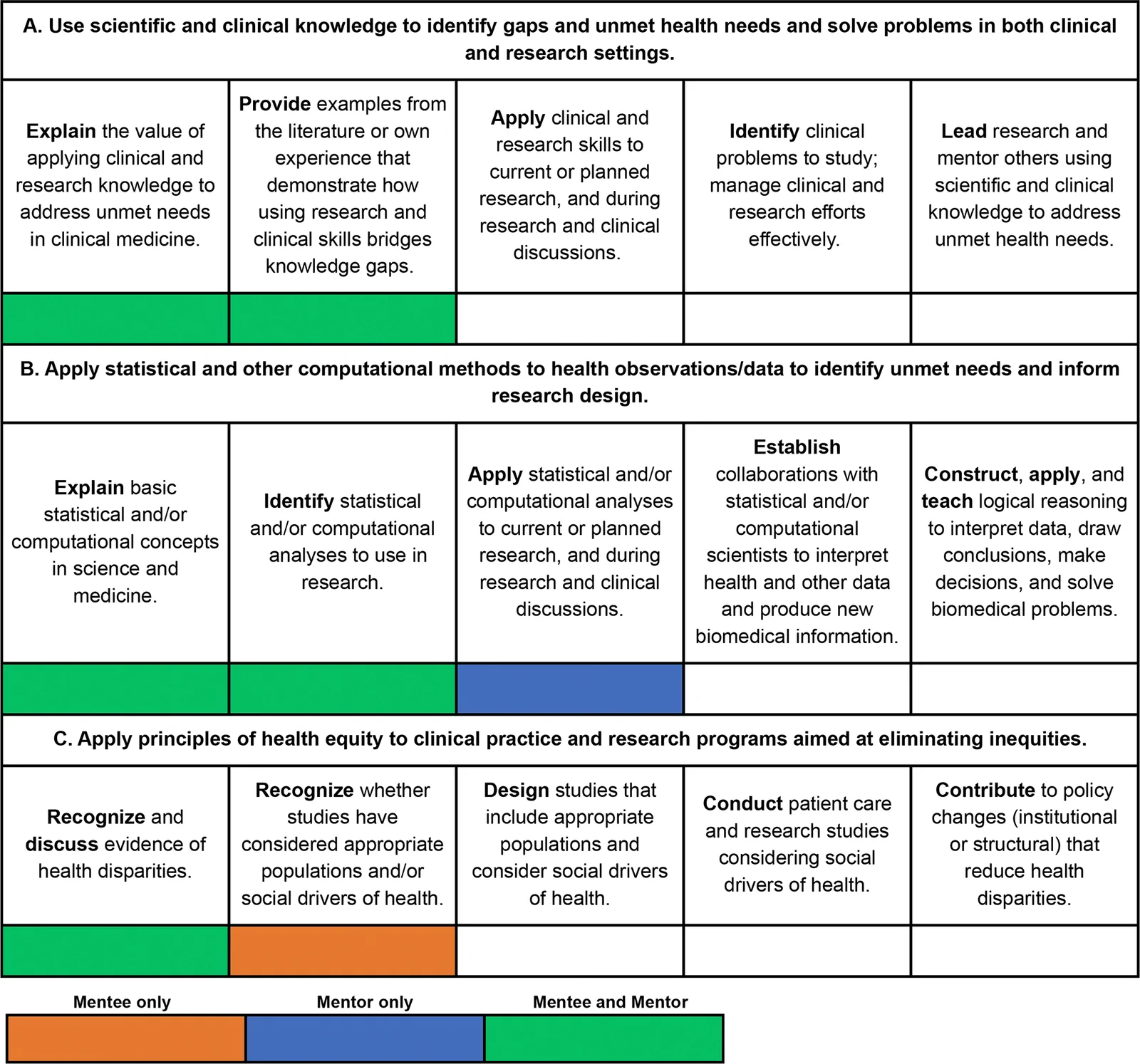
Possible report format comparing competency-based assessment by mentee and mentor and illustrating possible patterns of agreement on the MD-PhD Competency Toolkit, developed in 2022 by the MD-PhD Competencies Development Workgroup, part of the Association of American Medical Colleges Group on Research, Education, and Training. A. Mentee and mentor agree about the highest milestone mastered by the mentee. B. Mentor suggests that the mentee has mastered an additional milestone. C. Mentee suggests that they have mastered an additional milestone. Bold text indicates the primary behavior verb for each milestone.

**Table 1:** Competency domains, competencies, and developmental milestones on the MD-PhD Competency Toolkit, developed in 2022 by the MD-PhD Competencies Development Workgroup, Association of American Medical Colleges Group on Research, Education, and Training.^a^

Competency domain and competency	Milestones				
	Novice	Advanced beginner	Competent	Proficient	Expert
**Research to health**					
A. Use scientific and clinical knowledge to identify gaps and unmet health needs and solve problems in both clinical and research settings	**Explain** the value of applying clinical and research knowledge to address unmet needs in clinical medicine	**Provide** examples from the literature or own experience that demonstrate how using research and clinical skills bridges knowledge gaps	**Apply** clinical and research skills to current or planned research, and during research and clinical discussions	**Identify** clinical problems to study; **manage** clinical and research efforts effectively	**Lead** research and mentor others using scientific and clinical knowledge to address unmet health needs
B. Apply statistical and other computational methods to health observations and data to identify unmet needs and inform research design	**Explain** basic statistical and/or computational concepts in science and medicine	**Identify** statistical and/or computational analyses to use in research	**Apply** statistical and/or computational analyses to current or planned research and during research and clinical discussions	**Establish** collaborations with statistical and/or computational scientists to interpret health and other data and produce new biomedical information	**Construct**, **apply**, and **teach** logical reasoning to interpret data, draw conclusions, make decisions, and solve biomedical problems
C. Apply principles of health equity to clinical practice and research programs aimed at eliminating inequities	**Recognize** and **discuss** evidence of health disparities	**Recognize** whether studies have considered appropriate populations and/or social drivers of health	**Design** studies that include appropriate populations and consider social drivers of health	**Conduct** patient care and research studies considering social drivers of health	**Contribute** to policy changes (institutional or structural) that reduce health disparities
**Communication skills (written and oral)**					
A. Communicate scientific ideas and their application to clinical medicine to nonexpert audiences, clinicians, and scientists	**Follow** a structured format tailored to present fundamental concepts to each audience	**Communicate** fundamental concepts with few inaccuracies. **Recognize** limits of own understanding	**Accurately** communicate concepts, relationships, novel ideas, and/or findings, highlighting knowledge gaps	**Adapt** the design and communication style to the target audience to maximize engagement	**Coach** others to use best scientific communication practices
B. Communicate with funding agencies to leverage the dual expertise of physician-scientists	**Discuss** how research is funded using grants from federal, foundations, and bioindustry	**Develop** and/or **submit** predoctoral fellowship or grant application with mentor’s guidance	**Participate** in a postdoctoral grant application and review cycle with mentor’s guidance	**Develop** a funding application in response to a clinical knowledge gap with emerging independence	**Develop** and **lead** funded cross-disciplinary translational research programs
**Professional growth**					
A. Navigate differences in professional norms between clinical care and research settings during career transitions	**Describe** basic differences between clinical and research workplaces	**Navigate** across differences in professional norms in clinical and research settings with coaching	**Transition** between clinical and research workspaces with emerging independence	**Engage** in clinical and research duties independently while identifying strategies to maximize productivity	**Integrate** clinical and research activities in a way that maximizes complementarity and career satisfaction
B. Practice lifelong learning and growth mindset habits that support long-term engagement as a physician-scientist	**Describe** and explore opportunities that combine research and clinical expertise	**Seek** exposure to new research and clinical ideas while engaging deeply in curiosity-driven exploration	**Apply** experiences and evidence to inform clinical and research specialty choices	**Integrate** experiences to select a career path that maximizes potential, enables discovery, and promotes individual work-life satisfaction	**Model** lifelong learning and growth mindset habits to mentor rising physician-scientists
C. Use a mentor network to support your individual physician-scientist career pathway	**Describe** importance of mentors in career development and professional growth	**Identify** research and clinical mentors and assemble a team of advisors for specific needs; **engage** effectively as a mentee	**Differentiate** between the relative strengths and opportunities that individual mentors offer	**Assess** mentoring needs to optimize team of research and clinical mentors	**Adapt** mentoring team to meet evolving needs and career progression
D. Use mentoring skills to support the physician-scientist careers of others	**Define** the role of mentors and mentees in clinical and research settings	**Explore** the mentor role with junior trainees in clinical and research settings	**Compare** and **contrast** roles of coaches, sponsors, and mentors; **evaluate** own mentorship skills	**Adjust** mentoring style to address needs of mentees from diverse backgrounds	**Function** and **model** as an inclusive coach, and mentor to optimize trainee outcomes
E. Work in cross-disciplinary teams of clinicians, scientists, translational researchers, and other stakeholders	**Describe** the roles of different members of a cross-disciplinary team	**Work** effectively and collaboratively in a cross-disciplinary team	**Practice** and **reflect** on effective leadership skills for cross-disciplinary teams	**Critically** evaluate the roles of team members to enhance the team effectiveness	**Assemble** and **lead** teams that are diverse, effective, and cross-disciplinary
**Personal growth**					
A. Manage personal signs of stress or burnout during transitions between clinical and research settings.	**Recognize** resistance to feedback or disengagement from learning activities	**Plan** and **practice** strategies to promote personal wellness	**Accept** feedback and guidance to remain engaged in clinical and research activities	**Adapt** effective strategies to balance clinical and research duties during transitions	**Mentor** others about strategies to balance clinical and research duties during transitions
B. Practice persistence skills to remain in a career that utilizes the skills, knowledge and attributes from the dual degree	**Reluctant** to ask for help and resources	**Practice** resilience and **ask** for help and access resources	**Recognize** and **manage** expectations of career and personal demands	**Articulate** current and future needs for career and personal goals	**Define** personal success; **guide** others to articulate needs and goals
C. Develop personal values and identity and professional identity as a physician-scientist	**Describe** values of a physician-scientist	**Articulate** personal and professional values relating to identity as a physician-scientist	**Use** personal and professional values as a physician scientist in choosing a clinical specialty	**Integrate** personal and professional values as a physician-scientist during clinical training	**Model** personal and professional values for other physician scientists
D. Minimize the impact of personal biases in clinical practice and research study design	**Explain** how biases impact others’ views	**Recognize** personal biases and explore strategies to minimize their impact	**Implement** strategies to minimize impact of personal biases in the role of physician-scientist	**Advocate** for changes in policies and practices that perpetuate health inequities	**Lead** institutional or community efforts to change policies that perpetuate inequities

a Each row represents a distinct competency, and the corresponding milestone descriptions progress horizontally from left to right, reflecting increasing levels of autonomy and mastery. The milestones are intended to guide learners, mentors, and program directors in assessing growth across the continuum of physician-scientist training—from entry into an MD-PhD program (novice) through completion of training and transition to independent practice (expert). Bold text indicates the primary behavior verb for each milestone.

## Teaser text:

The MD-PhD Competencies Development Workgroup, part of the Association of American Medical Colleges Group on Research, Education, and Training, developed a community-informed toolkit that defines and assesses competencies essential to physician-scientist training. The MD-PhD Competencies Toolkit includes 14 competencies within 4 domains and milestone-based assessments to promote learner growth, mentoring, and program evaluation. This structured approach provides one common framework for programs nationwide to guide learners, align curricular goals, and support the next generation of physician-scientists, bridging the worlds of medicine and research.
